# No Need to Worry? Anxiety and Coping in the Entrepreneurship Process

**DOI:** 10.3389/fpsyg.2020.00398

**Published:** 2020-03-12

**Authors:** Neil A. Thompson, Marco van Gelderen, Laura Keppler

**Affiliations:** Department of Management and Organisation, Vrije Universiteit Amsterdam, Amsterdam, Netherlands

**Keywords:** entrepreneurship process, negative emotions, anxiety, coping, fear of failure

## Abstract

Understanding experiences of and responses to anxiety is foundational to developing robust theories of entrepreneurial behavior. Using open-ended, vignette and graphical elicitation interviews with 77 entrepreneurs, we inductively investigate the experience of and coping responses to anxiety during the entrepreneurship process. We develop a comprehensive and dynamic goal-striving model to explain experiencing and coping with entrepreneurial anxiety by integrating empirical findings with appraisal and control theories. In doing so, we theorize that entrepreneurial anxiety is endogenous to a cyclical conception of goal-striving, such that various sources of anxiety make sense only in consideration of the goals, standards or values to which they pertain. In this regard, entrepreneurs’ coping responses influence four different points of an iterative goal-striving cycle—an insight that moves beyond problematic static and binary coping classifications.

## Introduction

Entrepreneurship provides organizational psychologists a unique context wherein uncertainty, financial and personal exposure, and psychological ownership combine in a more extreme as well as isolated manner than found in large, mature organizations (Baron et al., 2007). While research on entrepreneurial employees argues that stress arises from role conflict, role ambiguity, and role overload (Dess, 2003), independent entrepreneurs often face additional financial, social and psychological uncertainties and risks that can cause stress and anxiety (Parslow et al., 2004; Rauch et al., 2018). As part of a broader research stream advancing “hot” theories of entrepreneurial emotions and well-being (Cardon et al., 2012; Shepherd, 2015; Stephan, 2018), we study the omnipresence of anxiety—worry, doubt and unease about something with an uncertain outcome (Miceli and Castelfranchi, 2005)—among entrepreneurs. As anxiety is experienced as unpleasant, contemporary research has been guided by the principle that it should be minimized to reduce its strain on decision-making abilities and effort (Grichnik et al., 2010; Welpe et al., 2012; Doern and Goss, 2014; Kollmann et al., 2017). However, recent studies have shown that anxiety may also facilitate the creative thinking and effort of entrepreneurs (Foo et al., 2009; Cacciotti et al., 2016). Moreover, although anxiety is ubiquitous and negatively experienced, entrepreneurs express satisfaction with their work (Benz and Frey, 2008; Stephan and Roesler, 2010; Morris et al., 2012; Barba-Sánchez and Atienza-Sahuquillo, 2017). This suggests that persisting entrepreneurs are often able to harness anxiety and thrive in these circumstances.

Nevertheless, our understanding of the ways in which entrepreneurs transform anxiety into positive behavioral outcomes remains incomplete. To date, anxiety is thought to arise from negative perceptions of environmental stimuli that threaten venture survival, giving rise to fear of failure (Cacciotti et al., 2016). However, anxiety may also arise when failure of the venture is not directly at stake. It is not only threats to business survival that surfaces anxiety, but to a range professional and personal goals and standards. In addition, when studying how entrepreneur deal with fear of failure, the extant literature has focused on problem and emotion-focused (Patzelt and Shepherd, 2011), or avoidance and approach-focused (Uy et al., 2013; Cacciotti et al., 2016) coping of entrepreneurs. As Folkman and Lazarus (1980) and Skinner et al. (2003)point out, binary and static coping categories are problematic because underlying coping behaviors often fit into both categories which undermines their explanatory power. It follows that there is a need to better unravel the dynamics and mitigation of anxiety to answer how entrepreneurs persist in the face of ubiquitously experienced anxiety.

To address these issues, we seek to inductively answer the question of how entrepreneurs experience and cope with anxiety during the entrepreneurship process in order to meet their goals and standards. We employ a qualitative methodology that combines two waves of open-ended and structured interviews with a total of 77 entrepreneurs. Through recursive data collection, analysis and links to control theory (Carver and Scheier, 1981, 1998) and appraisal theory (Lazarus, 1966, 1999; Lazarus and Folkman, 1984), we develop an goal-striving model of entrepreneurial anxiety and coping.

Our empirical findings and conceptual model contribute to the literature in a number of ways. First, we will go beyond implicit acknowledgment of the importance of goals in the entrepreneurship process (Patzelt and Shepherd, 2011; Jenkins et al., 2014; Cacciotti and Hayton, 2015). We will theorize that anxiety is endogenous to a cyclical conception of goal-striving, such that various sources of anxiety make sense only in consideration of the goals, standards or values to which they pertain. Anxiety occurs when entrepreneurs perceive of an altered situation and assess that it threatens the achievement of any of a variety of business or personal goals, standards or values. Secondly, we will study anxiety in both its valence and activation aspects. Whereas the experience is unpleasant (valence), entrepreneurs report that anxiety often makes them work harder and better (activation). The extant literature has predominantly focused on the inhibiting effects on fear of failure.

Thirdly, our conceptual and empirical work will show that coping categories influence four different points of an iterative goal-striving cycle—an insight that moves significantly beyond problematic static and binary coping classifications. To cope with anxiety, we reveal that entrepreneurs undertake behaviors corresponding to four coping categories: directly address the issue at hand, change perceptions, adapt goals, and increase coping ability. Subsequently, these four coping categories are shown to be used concurrently within a cyclical process of goal-striving that dissipates anxiety concomitantly with increased effort and satisfaction. Accordingly, our dynamic and comprehensive model explains both how and why entrepreneurs experience anxiety, as well as how and why they transform it into positive cognitive and behavioral outcomes.

As such, our study contributes to the anxiety and coping literatures more generally by allowing for the development of explanations and models from a context where uncertainty, challenges, financial and personal exposure, and psychological ownership combine in a more extreme manner than found in ordinary employment or private settings. Consequently, our study opens up new research questions, and has practical implications for entrepreneurial education and training.

## Theoretical Motivation and Opportunities for Conceptual Development

Entrepreneurship is widely perceived to be an “emotional rollercoaster” (Schindehutte et al., 2006) involving a range of positive and negative emotions (Fodor and Pintea, 2017). Although being an entrepreneur is experienced as satisfying (Benz and Frey, 2008; Morris et al., 2012; Stephan, 2018), entrepreneurs routinely face uncertainties, setbacks and challenges (van Gelderen, 2012). The negative emotions generated by the entrepreneurial process raise the question why and how surviving entrepreneurs are able to persist and even thrive under such conditions. Perseverance, resilience, and the ability to regulate emotions are seen as essential vital for entrepreneurial success (Millán et al., 2012; Holland and Garrett, 2015; Barba-Sánchez and Atienza-Sahuquillo, 2017; Chadwick and Raver, 2018; De Cock et al., 2019).

Recently, fear of failure has been a topic of study and is sometimes used synonymously with anxiety (Cacciotti and Hayton, 2015; Cacciotti et al., 2016). However, we follow Miceli and Castelfranchi’s (2005) argumentation that anxiety encompasses feelings of fear (of failure), doubt, worry, and unease. Fear (of failure) is just one form of anxiety, in that entrepreneurs are not only fearful of eventual business failure. Anxiety also includes worries about a range of much more proximal threats (e.g., pertaining to financial concerns, completing tasks, responsibility to others or maintaining positive self-image) and doubts about abilities to deal with situations effectively, even when the business is not at risk. In addition, other emotions than fear can also coincide with anxiety (e.g., shame and guilt). Put another way, as we will show in this article, many entrepreneurs experience anxiety without feeling fear (of failure). Furthermore, while fear always has an immediate and direct object, anxiety can be lingering and indeterminate. Hence, although overlapping, anxiety is a broader term and therefore the focus of this study.

Although the literature has made significant gains, opportunities for conceptual development remain. The first opportunity concerns the study of anxiety when in the entrepreneurial process, rather than as a deterrent to enter the entrepreneurial process. Negative emotions such as fear of failure have been found to be a deterrent to starting a business or acting on an opportunity (Grichnik et al., 2010; Ekore and Okekeocha, 2012; Podoynitsyna et al., 2012; Welpe et al., 2012; Doern and Goss, 2014; Kollmann et al., 2017). Grichnik et al. (2010) find evidence that fear negatively influences not only opportunity evaluation, but also opportunity exploitation. This is supported by Kollmann et al.’s (2017) experimental study that found the mere perception of obstacles activates a fear of failure, which, in turn, has a detrimental impact on opportunity evaluation and exploitation. However, it has recently been proposed that fear can actually be a motivator during the entrepreneurship process (Hayton and Cholakova, 2011). Drawing on interviews with 35 entrepreneurs, Cacciotti et al. (2016) find that fear of failure may lead to increased effort. Similarly, Foo et al. (2009) find evidence that negative valence associated with anxiety (e.g., upsetness, irritability, nervousness, distress, and jitteriness) positively predict the effort put toward tasks that require immediate attention. Given the empirical evidence that anxiety may facilitate or hinder the efforts of entrepreneurs, a number of scholars have called for more inductive investigations to explain the dynamics between negative affect and coping during the entrepreneurship process (Cardon et al., 2012; Cacciotti and Hayton, 2015; Grégoire et al., 2015; Shepherd, 2015).

The second opportunity revolves around anxiety being inherently tied to many goals and standards rather than mere venture survival. For example, Patzelt and Shepherd (2011), citing Folkman and Moskowitz (2004, p. 747), point out that “coping enables individuals to deal with negative emotions that arise when important goals have been harmed, lost, or threatened”. Similarly, Cacciotti and Hayton (2015, p. 165) posit that “the nature of fear and the diverse cognitive and behavioral mechanisms that it triggers suggests that it could be a friend as much as a foe, by causing greater striving toward desired goals.” Cacciotti et al. (2016) findings suggest anxiety can be related to multiple higher or lower-order goals and standards, which range from threats to achieving financial success, and maintaining self-esteem to completing everyday tasks. However, no explicit theorizing of the relation between goals and anxiety is provided.

The third opportunity involves expanding our understanding of coping during the entrepreneurship process, which currently remains limited. Existing research on the coping behaviors of entrepreneurs has highlighted their use of problem or emotion-focused coping (Patzelt and Shepherd, 2011) and avoidance or approach coping (Uy et al., 2013; Cacciotti et al., 2016) to reduce anxiety. Patzelt and Shepherd (2011) demonstrate that there is a negative relationship between self-employment and the experience of negative emotions, generally, and this relationship is stronger for those who use problem and emotion-focused coping than for those who do not. Uy et al. (2013) and Cacciotti et al. (2016) argue that using approach and avoidance coping—taking action or delaying action—helps entrepreneurs to maintain their wellbeing and overcome anxiety in the entrepreneurship process, particularly if they have prior entrepreneurial experience. Thus, in the entrepreneurship literature, coping responses continue to be viewed on aggregated levels, even though such binary and static classifications have been challenged in the mainstream coping literature. Skinner et al. (2003) point out that problem and emotion-based coping are not mutually exclusive and that most ways of coping can serve both functions and thus fit into both categories. Moreover, as stated by Lazarus (1996, p. 293), “although it is tempting to classify any coping thought or act as either problem-focused or emotion-focused, in reality any coping thought or act can serve both or perhaps many other functions.” Similarly, approach and avoidance are complementary coping processes and, over the course of dealing with taxing situations, people can—and usually do— repeatedly cycle between them (Gross, 2015). Finally, while the extant literature sees coping with negative affect such as anxiety as serving the function of reducing its aversive experience, we are interested in how anxiety may spur those who are actually committed to their venture on to perform at a higher level.

Accordingly, the critical problem for the field is to develop a more situated and dynamic understanding of anxiety and coping responses during the entrepreneurship process. In this study, we act on all three opportunities described above, and provide an empirical and theoretical answer to the question: *how do entrepreneurs experience and cope with anxiety during the entrepreneurship process in order to meet their goals and standards?*

## Methodology

Qualitative research is appropriate when the research question focuses on a process—or how something occurs—and when a theory needs to be developed or elaborated (Creswell and Miller, 2000). Given the ethical dilemmas of inducing anxiety in subjects in laboratory experiments, intensive interviews are the method of choice for researching this sensitive phenomenon. Under such circumstances, in-depth interviews are more likely to create original and precise accounts of previously unexplored phenomena (Grégoire et al., 2015; Shepherd, 2015). In particular, we draw on template analysis to inform our data collection techniques and to structure our data analysis (Brooks and King, 2014; King and Brooks, 2017).

### Research Design

Template analysis is commonly used in qualitative psychology (Kent, 2000; Poppleton et al., 2008), particularly in occupational health (Gollop et al., 2004; Brooks et al., 2015). We chose to use template analysis for two main reasons. First, it uses two waves of data collection to reveal and refine emergent patterns. Specifically, in the first wave of data collection, it allows us to inductively identify the sources and coping mechanisms in the initial startup of a venture through open and axial coding. Second, in the second wave of data collection, template analysis allows us to systematically assess and refine our findings over a longer period of time by collecting structured interview data with entrepreneurs within one to 5 years after foundation. Therefore, the core strength of template analysis is that researchers modify or elaborate upon emerging findings while paying attention to whether contradicting evidence can be found. In addition, template analysis provides a means to reach data saturation. In qualitative research, once research methods generate no new, additional and novel information, the researchers have reached saturation. We use template analysis to continue to collect data beyond saturation, in order to ensure the validity of findings. Finally, we use a qualitative research design instead of survey methods and existing scales as our aim is not to assess levels of anxiety in general and relate those to an outcome (success or failure). Instead, we are interested in anxiety insofar it is engendered by engaging in entrepreneurial activities, and then in particular its sources, immediate effects, and forms of coping when dealing with it. As our literature review reveals, we have little empirical research of anxiety in entrepreneurial settings, and there are no established measures available that would suit our purposes. Below the details of template analysis and the two waves are discussed in more detail.

### First Wave Sampling, Data Collection and Analysis

We used theoretical sampling to include entrepreneurs who are currently and actively engaged in entrepreneurship, who founded their business within the last 12 months and responded that they had or were experiencing anxiety. In order to optimize external validity, we cast a wide net to understand the various sources of anxieties, coping responses and their interaction by developing a website as a point of contact for entrepreneurs (*N* = 33). We sought a wide-range of respondents (in terms of age, gender, and nationality) with ventures of varying characteristics [in terms of solo or team, the age of the venture, the subjective stage of development, full-time freelance or company, the size (number of employees) and sectors]. Table 1 summarizes the characteristics of the entire sample.

**TABLE 1:** Descriptive sample statistics.

		**First wave (*N* = 33)**		**Second wave (*N* = 44)**		**Total (*N* = 77)**	
**Variable**	**Category**	***N***	**% of 33**	***N***	**% of 33**	***N***	**% of 77**
Gender	Male	22	67%	33	75%	55	71%
	Female	11	33%	11	25%	22	29%
Nationality	Dutch	20	61%	35	80%	55	71%
	Non-Dutch	13	39%	9	20%	22	29%
Freelancer	Yes	10	30%	13	30%	23	30%
	No	23	70%	31	70%	54	70%
Stage of development*	Nascent	10	30%	1	2%	11	14%
	Early growth	23	70%	8	18%	31	40%
	Established	0	0%	24	54%	24	31%
	Established + growth	0	0%	11	25%	11	14%
Sector*	Manufacturing	1	3%	1	2%	2	3%
	Retails	2	6%	11	25%	13	17%
	Business services	26	79%	18	41%	44	57%
	Consumer services	4	12%	14	32%	18	23%

	**First wave (*N* = 33)**			**Second wave (*N* = 44)**			**Total (*N* = 77)**		
**Variable**	***M***	***SD***	**Range**	***M***	***SD***	**Range**	***M***	***SD***	**Range**

Founder age*	31	8	23–59	39	12	20–62	35	11	20–62
Venture age* (months)	11	3	2–13	34	31	5–192	24	26	2–192
Employed (in fte’s)	3	3	1–12	21	76	1–500	13	58	1–500

* Difference (p < 0.05) between wave 1 and wave 2.

Open-ended questions were used to investigate the entrepreneurs’ various sources of anxiety, their immediate affective experience of anxiety, and their coping responses, with an average interview length of 90 min. We started out by asking broad, open-ended questions (“Tell me about your experiences with anxiety,” “What do you think made you feel this way?”, “How did you experience this anxiety and what effects did it have on you?”, “How did you cope with this anxiety?”). We followed up by asking for examples and probing their responses further. Given the sensitivity of the topic, interviews were conducted face-to-face, which is preferable when discussing emotionally sensitive experiences because the interviewer can react to visible cues and comfort the interviewee. In order to minimize response bias, we ensured confidentiality, encouraged interviewees to talk openly and unrestrained without passing judgment and to choose their own words to tell their personal story.

The interviews were recorded, transcribed and coded using open and axial coding (Guest et al., 2012). In the first phase, any source of anxiety, subjective experience, or coping response was assigned a code (indicated as a comment on Microsoft Word) using an open-coding technique. First-order coding adhered closely to the respondents’ vocabulary and terminology, and involved limited interpretation or evaluation. Each co-author independently created first-order codes corresponding to cause, effect and coping response type. A process of consensual coding (Guest et al., 2012) was employed to resolve any disagreements about codes. Each time the coders reached a point where their coding did not agree, the reasons for the discrepancy were discussed, a solution was agreed on, and codes were revised if necessary. This process resulted in 274 (sources), 120 (subjective experience), and 319 (coping) first-order codes. Next, looking for repetition and commonality using axial coding, the first-order codes were grouped together in Microsoft Excel based on response type until a limited number of higher, second-order codes emerged—50 (sources), 20 (subjective experience), and 33 (coping). These codes were again grouped and labeled according to response type—10 (sources), 4 (subjective experience) and 8 (coping) to complete an initial template, which guided our next wave of data collection.

### Second Wave Sampling, Data Collection and Analysis

In line with template analysis, we collected a second wave of data. Whereas the first wave helped us to exploratively derive categories of sources, subjective experience, and coping, the second wave was used to establish the prevalence rate of these categories. Moreover, we now sampled somewhat older firms, between 1 and 5 years old, so that we could track developments in anxiety sources, experiences and coping over a longer time period. Thus, using theoretical sampling (*N* = 44), we selected entrepreneurs who were currently and actively engaged in entrepreneurship, responded that they had or were experiencing anxiety and whose businesses were founded between 1 and 5 years ago. Again, we sought a wide variety of entrepreneurs in terms of personal and venture characteristics (see Table 1). Chi-square and *t*-tests showed that the two samples differed in the age of the entrepreneur, and the age and stage of development of the venture, which aligns with the different sampling criteria used for the two different waves. The second wave had a more representative distributions of sectors, as the aim of the second wave was to validate categories of anxiety sources, experiences, and coping responses, as well as their prevalence rates. Using our template from the first wave of data, we developed a structured interview protocol using two interviewing techniques—vignette (Jennings et al., 2015; van Gelderen, 2016) and graphic elicitation (Crilly et al., 2006; van Gelderen, 2016). These techniques also helped to minimize retrospective and response biases of open-ended interviews by anchoring and eliciting more detailed responses in relation to given scenarios.

#### Vignette Technique

The vignette technique elicits perceptions, opinions, beliefs, and attitudes from respondents as they comment on short stories depicting realistic scenarios, thus it allows us to establish prevalence rates. We created 10 hypothetical vignettes (see Supplementary Appendix 1 for complete overview) corresponding to the 10 sources of anxiety derived from the initial template. Respondents were shown a vignette and then asked if they had experienced anything similar during their entrepreneurial experience. If a respondent had not experienced anything similar, then we moved on to the next vignette. If a respondent had experienced something similar, the respondent was encouraged to share examples and details from his or her own experience.

#### Graphic Elicitation Technique

**FIGURE 1:**
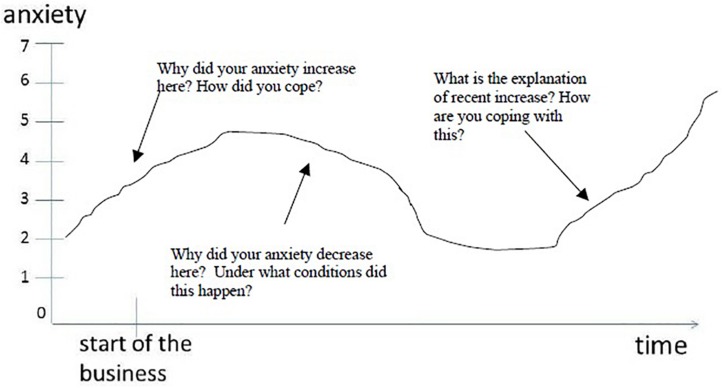
Example of elicitation graph.

Additionally, we followed up with a graphic elicitation interviewing technique to collect fine-grained data about their experience, specifically focusing on the coping mechanism(s) they used in response. In this technique, each vignette was accompanied by a graph in which time runs along the *x*-axis, starting with venture founding and ending with the present. The *y*-axis represents the level of anxiety, starting with a complete lack of anxiety (0) to fully experiencing this type of anxiety (+7). The respondent was asked to draw a line on the graph depicting the intensity of anxiety with regard to that specific vignette (source of anxiety). Figure 1 below provides an example.

Importantly, this technique was not used deductively as a general measure respondents’ experience and coping abilities with anxiety over time, but was rather used to elicit more fine-grained interview data. After participants drew a line, we pointed to different fluctuations in the line asking the respondent questions such as: “What factors explain any curves or changes in the line?”, “When/under what conditions did this happen?”, “How did you experience anxiety during this period?”, “How did you cope with the anxiety?”, “Were there any positive aspects to this experience of anxiety?”, and further, probing questions.

Following the completion of each vignette and graphic elicitation techniques, we ended the interview session with an open-ended question asking if there were any sources of anxiety or coping responses from the entrepreneurs’ experience that we had not covered. We, again, coded responses by response type, using consensual and hierarchical coding following the same procedure in the first wave. This led to 529 (sources), 211 (subjective experience), and 452 (coping) first-order codes, grouped into 59 (sources), 52 (subjective experience), and 46 (coping) second-order codes, respectively. Finally, the second-order codes were matched to themes (10 sources, 4 subjective experience and 4 coping) corresponding to the initial template or a modified template when necessary (coping codes were specifically narrowed to 4), and prevalence rates were calculated based on yes/no responses to vignettes (for sources).

## Findings

In this section, we provide an empirical and theoretical answer to the question how and why entrepreneurs experience and cope with anxiety during the entrepreneurship process in order to meet their goals and standards. To do so, we first report the sources of anxiety: those factors, situations and conditions that represent a threat to goals and standards. In the next sub section, we discuss the experience and immediate effects of anxiety. We then turn to specifying four categories of coping behaviors that entrepreneurs employ when experiencing anxiety, and their cyclical, iterative use. In the fourth and final sub section of this chapter, we discuss patterns in how anxiety develops over time. Altogether, the findings inform our conceptual model in the chapter 5.

### Goal-Striving and Sources of Anxiety

In Tables 2, 3 we report the higher-order sources of anxiety as revealed from our analysis. Table 2 presents codes and themes, Table 3 representative quotations. Overall, we find that anxiety is not only caused by immediate threats to a business’ survival, as a strict focus on fear of failure would have implied. Sources of anxiety are related to various higher and lower-order personal and professional goals, standards and values. As such, sources of anxieties derive from multiple and simultaneous goals, and the goal of successfully starting a new venture is coupled with a range of values. Please note that goals have a hierarchical relationship to one another (e.g., high-order venture success versus sub-goals of pitching a venture idea) (Austin and Vancouver, 1996), with the salience and ordering of goals varying from person to person.

**TABLE 2:** Sources of anxiety – data structure (final template).

**Response type**	**First order codes (examples)**	**Second order codes**	**Themes**	**Prevalence rate (only second wave)**
Source of anxiety	Doubts gap between supply and demand; doubts about price and demand; doubt if concept will work/value; worries about business concept	Business concept; unclear problem; product failure	Business concept viability	45.5%
	Investing more for growth; new growth issues, space and collaborations; not growing fast enough; unsure about how to scale, go to next level; finding, pitching to investors; uncertain when to approach VC	Growth, investing more; growth, new issues; growth, speed of; growth, acquisition	Growth	47.7%
	Wanted to get a higher education; doubt if should have done traineeship; doubt decision about other job opportunities; not participating in other obligations; less time for other things, girlfriend; no time for friends; not enough time to pursue all interests	Threat to livelihood; return to wage employment	Opportunity costs	38.6%
	Doubt if working hard enough; doubting choices; not having the right skills; doubt capabilities to fix problem; Realizing not good at task; doubt negotiation abilities	Lack of experience and knowledge; self-doubt capabilities and effort	Capability	70.5%
	Dependence on one big client, no power; dependence on clients to pay on time; dependence on employees/interns; dependence, even though freelancer; getting steady supply, quality; dedication of collaborators; depending on partner to be accountant	Dependence on unreliable or few clients, supplier, partner, advertisers, and team	Dependence	75%
	Responsibility to co-founder; responsibility to pay salary, expectations; responsibility to other families; meet client expectations, responsible; responsibility to family, supporters	Responsibility toward client, supporters, team, and employees	Responsibility	52.3%
	Increased competition, uncertainty; worries about unknown, richer competitor; more experienced competition; doubt will compete with big companies; lack of fairness in market; inflation, currency, interest rates; worries about current politics	Competition; macroeconomic and political environment	Environmental uncertainty	59.1%
	Venture finances, not enough, too much; worries about case flow; worries about debt, restricting freedom; investment or paying rent; financial obligations, loan repayment	Finances, repaying loan; finances, cash flow; finances, debt	Finance	72.7%
	Being seen as arrogant, misperceptions; losses are public, perception of loss; deputation damage; loss of status; not being seen as professional; making things look better than they are; not being taken seriously, approval; what other people think and say; public presentation	Possible loss of status or reputation; image of self does not align with public image; exposure to public scrutiny	Social-appraisal	50%
	Not meeting high expectations of self; loss self-image of success; worries about self-esteem, personal failure	Loss of self-image as success; threat to social esteem	Self-appraisal	43.2%

**TABLE 3:** Sources of anxiety.

**Source of anxiety**	**Representative quotations**
Business concept viability	“The worry about the business concept is: can you make yourself known enough so that you have a steady flow of work overtime? I think that is where my anxiety is.” “It’s always the same; fear. First fear for viability in general. Do I have a viable solution?”
Growth	“We spent 9 months in 2012 during the economic crisis persuading people to invest in us, this brings a very high anxiety level.” “You get a pretty steep anxiety increase at the time that you have to think about scaling the business.”
Opportunity costs	“It takes time I could have spent with my family. It takes time I could have spent with my friends of from my social life.” “The business might not turn out to be as a success so, you put more time in it. But at the same time you cannot be the father you want to be.”
Capability	“I had to tackle situations where I had not much experience with. It made doubt my capabilities.” “Often I am in situations where I don’t have enough knowledge of, or don’t have the right capabilities.” “Being an entrepreneur is a constant internal discussion with regards to am I doing the right thing? Shall I invest, or not?”
Dependence	“I have to trust in [employee], that he does his work properly. The decrease of control increases as your company grows, and that brings anxiety.” “We are only with a few people, and I worry that then if one leaves it is more a problem.”
Responsibility	“People are actually dependent on me doing those tasks within a certain period of time. I try to get them as soon as possible, but if they are big tasks that can make me really anxious.” “In my head, I totally freaked out…we worked very hard and in the end we need to tell our client the big disappointment that their event is not happening. It was not our company, it was about disappointing our client.”
Environmental uncertainty	“The main anxiety and concern is when I have a new competitor, who will change the rules in the market. Then, I have to adapt myself while I don’t know exactly what is going to happen in the long term.” “2008 was the best year until then, and then the crisis hit. People stopped buying products. It is unpredictable.”
Finance	“It is not being anxious that what I am doing is not going to work, but it is going to enough money that I can live on it.” “Worries about money and if it doesn’t come in, what then? What happens, how do I pay the bills?”
Social appraisal	“There is an anxiety of how others expected me to perform. No matter what I achieve, there is always another higher expectation.” “The fact is that I had the feeling that I couldn’t meet the expectations other people had of me.”
Self-appraisal	“I think in the end it is about yourself, because it is never good enough in your own eyes.” “Last week I had 3 offers declined on 1 day. That was hard. It felt as a disappointment to myself. If the business would fail, that would be a personal failure.”

### The Experience and Immediate Effects of Anxiety

It is important to first distinguish between how anxiety is experienced, in other words its affective tone (valence), and its effects (activation) (Foo et al., 2015). Out of the combined 92 first-order codes in the first and second waves of data, 83 (90%) pertain to the negative *experiences* of the cognitive, emotional and physical symptoms of anxiety (see Table 4 for overview). While the subjective experience (valence) of anxiety is aversive, out of the 56 first-order codes pertaining to cognitive and behavioral activation because of anxiety, 42 (75%) refer to beneficial effects, such as being more adaptable, alert, aware, creative, active, driven, smarter, focused, reflective and bold. By contrast, only 25% of first-order codes concern instances in which the participants reported anxieties (temporarily) impaired their performance; for example, because of emotional exhaustion or decision paralysis. Accordingly, this suggests that anxieties often lead to enhanced cognitive capacities, which is in line with the findings of Cacciotti et al. (2016). Several respondents even stated that experiencing anxiety and feeling activated by it is the essence of being an entrepreneur.

**TABLE 4:** Immediate effects of anxiety – data structure (final template).

**Response type**	**First order codes (examples)**	**Second order codes**	**Themes**	**Prevalence rate (only 2nd wave)**
Valence and activation of anxiety	Alertness; fun; independence; joy, when overcome; aware; self-knowledge; work smarter;	Activating, alert, and stimulating effect	Positive cognitive effects	89%
	Innovative; adaptable; activated; creative; work harder	Proactive, innovative and adaptable	Positive behavioral effects	51%
	Being stabbed; bubbles up in belly; orange in stomach; weakness in legs; drinking alcohol; eating poor food; feel terrible, sick, headache; sleeplessness, tired	Negative effects on body; unhelpful behaviors	Negative physical experience of anxiety	35%
	Blameworthy; swearing; negative circular thoughts; panic; debilitating; overwhelmed; disappointment; loneliness; irritable; helplessness; impatience; loss of passion; aggressiveness; dejection; escalations in private life	Negative thoughts; negative emotions; loss of positive outlook	Negative cognitive and emotional experience of anxiety	68%

### Categories of Coping Responses

Our analysis inductively arrived at four categories of coping responses: directly influencing the situation at hand, changing the way the issue is perceived, adapting the goal or standard involved, or increasing coping options. In Tables 5, 6 we report the higher-order categories of coping and representative quotations. As these categories are relevant for every source of anxiety, we discuss coping responses in general, rather than in relation to each separate source of anxiety.

**TABLE 5:** Coping response categories – data structure (final template).

**Response type**	**First order codes (examples)**	**Second order codes**	**Themes**	**Prevalence rate (only 2nd wave)**
Coping with anxiety	Obtain information; improve aspects of venture; solve issues; discuss issues; plan, prioritize; change approach; increase effort; delay action; seek help	Planning; obtain information; increase effort; seek help	Directly influence the issue at hand	86.3%
	Invoke wider, long-term view; focus on positive aspects; manage perception of others; acceptance; pretend it is not there/denial; attribute to unstable or external cause; avoid negative comparisons	Optimism; long-term view; acceptance	Influence perceptions	75%
	Frame as learning goal; create sub-goals, intermediate goals; seek challenge; scale back goals; flexible goals; give up	Learning goal; create new (sub) goals; scale back goals	Involve the goal	54.5%
	Distraction and relaxation; meditation; seek social support; distancing (various forms); take time to reflect; increase long-term professional capability; self-affirmation; live healthier; turn to religion	Distance and relaxation; social support; physical health and personal well-being	Increase coping ability	70.5%

**TABLE 6:** Categories of coping responses.

**Categories**	**Representative quotations**
Cat. 1 Directly influencing the issue at hand	“If it is something that I can actually solve and think it is nice to solve, but I still have to think of ways, it just sticks with me until I solve it and that tends to be the middle of the night.” “I stayed in that situation for a while of going in that spiral of what to do and what to do. Then I put on the action mode and actually did things to solve the problem.” “I was concentrated to solve the problem as soon as possible. I tried to do everything that was in my power, maybe even a bit more.” “At some point, you have to decide on something that is going to stay stable otherwise you go crazy. That also gives you feeling of confidence and security.” “Start to try find people when you find someone, he solves your problem and [anxiety] goes back to the level that you don’t realize it.”
Cat. 2 Influence perceptions	“It takes a different mindset, but knowing that you expect the worst, you operate from that.” “Sometimes it helps to think about the worst that can happen. Okay, I lose my house, I lose everything, but, well, it sounds stupid, but it is still not the end of the world.” “If my project fails, my project fails, not that I fail. I’m of course emotionally bound to it, it is my baby to some extent, but if it fails, it fails and I still continue and I’m still myself.” “On a moment of doubt, you might only see the barriers on the road, and things get very negative. It is good to be very clear about the dangers and the negative sides, but also to see what you have achieved.”
Cat. 3 Involve the goal	“You should fail, because then you learn. That is the whole idea of being an entrepreneur.” “For me the only way to cope with it is by setting milestones. Saying, ‘if we don’t reach this barrier, we are going to stop.”’ “I look back at [project] as a big learning experience where I tried something that has been in my mind, I did it, it worked out differently than I had expected but I tried it.” “I’ve become much more realistic and I’m way more healthy about what success is about and that it’s not only about achieving the end goal or the intermediate goal but it’s also about how you do it, what is reasonable after a certain moment of time.”
Cat. 4 Increase coping ability	“For me, the more space I give myself, the quicker I get better and get more space in my head to figure something out.” “It is bringing in the balance. So, I make sure I do spend enough time with my family. But also religion. You make sure you have enough counterweight so the worries don’t go off the charts.” “I just sit down and try to relax, think about nothing and do nothing.” “You discuss the doubts you have. You need other people around you. You need to express yourself. If you just keep your thoughts to yourself, you will start thinking in circles.”

#### Category 1: Coping Responses That Directly Influence the Issue at Hand

The first category comprises coping responses that aim to eliminate the source of anxiety. Entrepreneurs cope by changing the actual situation so as to reduce the discrepancy between current situation and goals, standards, or value. This is regularly mentioned in a generic sense (solve issues, change approach, and increase effort) or in a specific reference to an aspect of the venture (reduce dependencies, cut costs, and improve the business model). For example, one participant said, “it annoys me sometimes that I am scared of things, then I push myself, just get over it, and do it. Even if I don’t like it so much.” As this example indicates, by stepping up performance, the entrepreneur addresses the issue at hand, and the anxiety which accompanies it reduces. The most often mentioned responses include seeking more information, making a plan and prioritizing efforts to eliminate the issue. For example, one participant stated, “when you make strategic decisions—you go left or right—it gets [your] faith up again and that’s how you get rid of the anxiety. You are constantly going through barriers by making up creative solutions.” Additionally, other individuals may be called on to help solve the issue at hand, such as the hiring of a lawyer in light of a lawsuit. Yet another response is restraint coping, which is described an expectation of a change in the situation that drives waiting for the underlying issue to subside (“sometimes you cannot do more, you just have to wait”).

#### Category 2: Coping Responses That Affect the Way the Issue Is Perceived

Coping responses that involve the subjective perception of the issue at hand, while leaving the environment and the goal unchanged, are outlined in the second category. The response with the highest frequency of occurrence in this category to adopt a long-term or broader view of situations. In the coping literature this is referred to as cognitive reappraisal, reframing or restructuring (Skinner et al., 2003; Gross, 2015). For example, when faced with the loss of a client, one participant mentioned, “I learned that I should be happy with myself and my accomplishments, independent of the results. I changed the image of myself and how others looked at me. I am now able to let it go.” Entrepreneurs also described their attempts to transcend the effects of immediate stimuli by bringing their attention back to their overarching goals. The threats to goals and standards and the accompanying anxiety made entrepreneur more reflective and caused them to rethink situations. This response specifically leads to subsequent reappraisals that focus on the positive aspects of the threat and encourages a hopeful outlook about them: “[anxiety] makes you think, and re-think things. Looking from different angles at things is a very positive thing.” Another coping response is to reframe threats through optimistic attribution. As one respondent explained, “The main feeling is that ‘I’ll figure something out.’ That’s why you become an entrepreneur—you believe that you can fix it.” By being optimistic, goal achievement or standard maintenance is continuous to be seen as feasible. Anxiety can be further reduced by attributing threats to external and transient factors, rather than internal and stable ones.

#### Category 3: Coping Responses Involving the Goal

In addition to tackling the sources of anxiety and thinking about them in new ways, entrepreneurs may turn to adapting their goals to alleviate anxieties. Goals, standards, and reference values have various applications in coping responses as they provide opportunities to reduce the discrepancy between the current state and the ideal state of goal achievement. The threats to goals and standards and the accompanying anxiety made entrepreneur more reflective and caused them to rethink their goals in terms of scale, scope, object, and timing. Framing goals as learning goals rather than performance goals (Kaplan and Maehr, 2007) is the most commonly reported response to reducing anxieties. One respondent explained, “If [the company fails], I would not consider that a failure. I would look back at it as a big learning experience where I tried something that had been on my mind, I did it, it worked out differently than I had expected, but I tried it. And I didn’t let it go.” The difference between learning and performance goals lies in the role ascribed to failure: failure makes it more difficult to reach a performance goal, but can actually enhance learning (Sitkin, 1992; Cope and Watts, 2000). Thus, a new learning goal is a sub-goal that could alleviate anxieties and enable the achievement of overall performance goals. Another strategy to reduce anxiety occurs if larger goals are broken down into these sub-goals that add lower layers to the goal hierarchy (Austin and Vancouver, 1996), which makes it clear on a more detailed level what is needed to reach the goal and can even highlight alternative ways to reach it. A third coping response in this category is to scale back goals in order to reduce anxieties. Goals can be scaled back in various ways, including time (taking longer to reach a goal), resources (starting with less resources than hoped for), and geography (a reduced geographical market area).

#### Category 4: Coping Responses That Serve to Increase Coping Options

The group of responses in this category is of particular importance as these were the responses entrepreneurs would turn to if they did not yet feel capable to solve the situation (cat.1), or reassess their assessment (cat.2), or their goals (cat.3). They would first need to work on their ability to do so. These responses should not be classified as avoidance coping, because the goal of these responses is eventually to be able to provide a cat.1, 2, or 3 response. For example, entrepreneurs who feel exhausted from anxiety may seek out ways to recharge, such as through sleep, social activities or exercise. Baumeister et al. (2006) discuss self-regulatory strength as a resource that becomes depleted after each use. After some form of relaxation or distraction self-regulatory strength is replenished and the entrepreneur may feel more able to directly target the source of their anxiety or to take a different perspective on the situation or on the goals that he or she is aiming to achieve. However, while distraction and relaxation are the most mentioned coping responses and can be an effective strategy, respondents pointed out the possible downside of them becoming habit-forming and harmful (i.e., continued elevated use of drugs and alcohol). Another response that can help entrepreneurs regain self-regulatory strength is seeking social support. This can take various forms, such moral support from a trusted mentor or partner, and can help boost or regain confidence. For example, one participant said, “I talk to my boyfriend; he is also an entrepreneur. He also understands a lot of what I have been going through. I talk to him and he calms me down.” In chapter 5, drawing on control theory and appraisal theory, we will connect the four different coping response categories outlined in this Section “Categories of Coping Responses,” by mapping them onto the goal striving cycle.

### Anxiety Dynamics

The graphic elicitation technique asked respondents in the second wave to track their anxiety levels with regard to each source from the pre-startup phase up to the present moment. We find that sources of anxiety need to be regarded in both a short and a long-term time frame. In terms of anxiety levels, we observed a large amount of variation *between* the different higher-level sources of anxiety, as well as *within* each source of anxiety. Levels of anxiety changed over time in a variety of patterns. Anxiety, for instance, was reported to be gradually increasing or decreasing, highly fluctuating, staying at even high or low levels, or fluctuating around even levels. Nevertheless, we were able to discern three main patterns from the inductive coding. First, fluctuations (sharp increase followed by decrease) typically occurred around significant short-term events (e.g., worries about business concept viability just before product or service launch). Second, longer-term gradual decreases were largely considered to be a function of experience, defined as a gradual improvement in coping responses and, as a consequence, reduced appraisals of threat. For example, worries about capability tended to dissipate with time as entrepreneurs report that they gained experience in coping with anxiety-provoking situations. Third, longer-term gradual increases in different sources of anxiety were related to growth of the venture. For these entrepreneurs, anxiety was low in the early startup phase, but increasing resource needs of the venture was accompanied with increasing anxiety (e.g., new dependence worries by hiring more employees or needing another round of investment). In the next section, we present our dynamic model of anxiety and coping in entrepreneurship that explains our findings.

## A Dynamic Model of Entrepreneurial Anxiety and Coping

**FIGURE 2:**
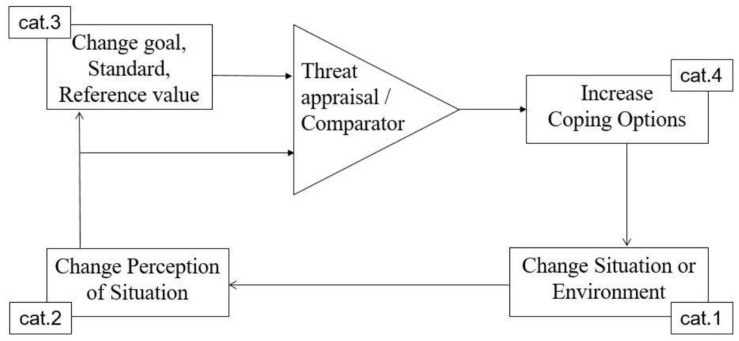
Anxiety and coping in the entrepreneurial goal-striving cycle.

The findings up to this point detail the various goal-related anxieties and coping responses of entrepreneurs that emerged inductively from our template analyses. Drawing on our temporal-oriented data acquired using the graphical elicitation technique, we present a conceptual model of anxiety and coping in entrepreneurship in Figure 2. Specifically, our model, which integrates control (Carver and Scheier, 1981) and appraisal theory (Lazarus, 1966), considers the emergence of, and coping with anxiety as an inherent part of the goal-striving cycle. Respondents use multiple responses in multiple categories going through the cycle various times, particularly when aiming to arrive at structural longer-term solutions with regard to sources of anxiety.

Control theory (Carver and Scheier, 1981, 1998) and appraisal theory (Lazarus, 1966, 1999; Lazarus and Folkman, 1984) argue that people continually face negative emotions as a result of threats to goals, and as such, they inform us as to how anxieties may be dealt with in entrepreneurial situations. The core idea of control theory is that self-regulation of behavior is enabled through a negative feedback cycle (Carver and Scheier, 1981; Vancouver and Day, 2005), which consists of recurrent comparisons between current situation and goals. Anxiety and coping are endogenous to the goal-striving cycle. The cycle begins with changes to the entrepreneurs’ situation (e.g., entrance of a competitor, need for more financial resources, a customer who cancels an order), followed by an entrepreneur’s perception of this changed situation. Perceptions are followed by an assessment of whether it affects the overall achievement of various goals and standards. This assessment, known as “comparator” in control theory (Carver and Scheier, 1998), or “primary appraisal” in appraisal theory (Lazarus, 1999), requires an appraisal of the threat, harm or challenge. If the new situation is seen as a threat to the goal hierarchy of the entrepreneur, s(he) assesses the available options of dealing with this issue, a process called “secondary appraisal” in appraisal theory. If no final solutions are immediately and easily available, this discrepancy will manifest itself as anxiety. The entrepreneur may then enact any of the responses in the coping categories outlined in the previous section (the boxes in Figure 2) with the aim to reduce the situation-goal discrepancy that gives rise to anxiety. Following enactment of coping responses, the cycle continues by entrepreneur’s new comparison (comparator) of the current and the desired state. If this renewed assessment concludes that no threat to goals exists, anxiety dissipates concomitantly. If not, then anxiety persists and again coping responses from any categories may be enacted, again feeding back into another round of appraisals. The same cyclical process applies also if the source of anxiety concerns a maintenance goal (or anti-goal, in control theory terms), such as maintaining a certain level of self-esteem. The difference is that the goal striving cycle is now discrepancy-enlarging rather than reducing, as one tries to steer away from the anti-goal (in this case, low self-esteem).

### Illustrative Case Examples

To illustrate our conceptual model, we provide two case examples of entrepreneurs from the sample; an inexperienced entrepreneur from wave 1 of our research design, and an experienced entrepreneur from wave 2.

#### Inexperienced Entrepreneur

Mark is a young co-founder of a new software company working to complete and test a “minimum viable product” (online platform) as soon as possible. One day, Mark unexpectedly finds another entrepreneur who, only days before, launched an online platform very similar to his and whom he had no idea existed previously. To appraise the situation, Mark immediately went to the competitor’s website, made a profile and tested its functionality. Ultimately, he determined it was a high-quality platform, which he described, “[Made me feel] really discouraged and my motivation went down…you have a pressure in your head.” Mark explained that he felt anxiety due to an inability to easily change the situation, a sense of not completing the “minimum viable product” task quickly enough (threat to concept viability), and feeling responsible for the wellbeing of his team.

Over the course of a month, Mark tried a number of different coping responses. First, he attempted to change his perception of the situation [cat. 2] by revisiting the competitor’s website “to find the bright side” a few days later. This led him to believe that the competitor’s platform was “completely unintuitive…I saw I could beat them. Also, if [it] works successfully, I [can] see that the market needs [my product] and the concept can be successful.” While this helped to reduce discrepancy, thus reducing anxiety, he continued to think, “everything went through my mind: ‘What should we do?’, ‘Is my crew in danger?’, ‘Should I have predicted it?”’ Mark then turned his attention to a start-up festival he had committed to organizing, which required dropping two courses at university and putting in no work toward the venture for 2 weeks. This distraction from the situation [cat. 4] reduced his anxiety temporarily; however, it only increased the worry that he was letting his team down. As he explained, “You are the founder of the start-up. So, you should be the person that works the most. But you don’t want to lie to them and say that you did something in that period.” Following the start-up festival, Mark met with the team and developed a new learning goal [cat. 3], agreeing that “it’s only [our] first start-up, you learn a lot from it. Everything is a huge experience and you learn a lot and it is a great reference for you that you had a startup.” Finally, Mark took action targeted at the environment [cat. 1] by: formally registering the business with the Chamber of Commerce, which made the venture “feel more tangible, now it feels like it can work, more real”; opening up to employees to ask their opinion on what they thought they should do; and making a strategic plan. These responses changed the subsequent appraisal of possible threats to the goals of the venture, which helped Mark reduce his anxiety levels and increase his motivation and effort in the further development of the product and business.

#### Experienced Entrepreneur

Francesca is a 53-year-old serial entrepreneur who co-founded a growing, 4-year-old business-consulting venture. The company’s founders decided that to grow they needed considerable financial investment and, in order to stay independent from external parties, the entrepreneurs decided to invest a large portion of their personal savings to finance the expansion. One day, Francesca was helping to register for a trademark, something she had little experience doing, when she received an email from another company’s lawyer requiring an immediate response. If Francesca failed to reply in 4 h, a team of lawyers would file a lawsuit. Francesca, whose co-founders were both away on vacation at the time said, “I did not understand it or the context; it was in English, it was difficult, it was a world that I did not understand and [I was] alone.” Francesca explained that she worried not only about her inability to complete the task, but also that the likely expensive litigation would result in compromising the overarching goal of the business succeeding, as well as the anti-goals of avoiding losing her personal and colleagues’ financial investment. In response to this anxiety, she took immediate action by seeking help [cat. 1] from a consultant to navigate a reply. She explained that she learned through her experience that “it is wise to understand, as an entrepreneur, you cannot do everything and that sometimes you just need a consultant.” Francesca explained that she also managed this, and other episodes of anxiety, by constantly maintaining her coping ability [cat. 4], describing how, “in the beginning, anxiety just happens to you, but later in your life you are more aware [of it]…and I personally build (counteracting habits) into my daily routine.” Francesca further said, “I go to bed early and eat healthier and do not drink alcohol and get up later and play more sports and collect people around me.” She also constantly manages her perceptions [cat. 2] as a way to limit anxieties before and after they arise. She primarily does this by keeping a diary, which, as she described, helps “you see how over the years a problem that, 10 years ago, made you lose sleep is actually nothing…writing history [allows you to] look back and reflect and learn from your brilliant failures. It also gets your worries out of your head and move it to your paper and recognize it.” Using these coping responses, Francesca was able to successfully avoid litigation, reduce her anxiety and harness its positive cognitive and behavioral effects.

## Discussion

In this study, we investigated the dynamics of anxiety and coping during the entrepreneurship process. We will now first discuss how our empirical and conceptual work contributes to the entrepreneurship literature on negative emotions and emotional self-regulation.

### Contributions and Integration

A first contribution is to expand theories of entrepreneurial anxiety and coping by grounding them in goal-striving behavior (Carver and Scheier, 1981, 1998; Lazarus, 1999; Skinner et al., 2003; Gross, 2015). Existing entrepreneurship research conceives of fear and anxiety as arising from subjective perceptions of environmental stimuli (Cacciotti et al., 2016), but do so without formalizing the role of goals and standards. Furthermore, the fear of failure literature implicitly assumes that business survival is assumed to be the only goal (Patzelt and Shepherd, 2011; Jenkins et al., 2014; Cacciotti and Hayton, 2015). Our study reveals that anxieties are intimately related to a range of goals and standards, the importance and order of which varies from person to person. These goals may or may not be explicit motives to start and operate an venture, but are nevertheless implicated [see Kehr’s (2004) distinction between explicit and implicit motives]. Anxiety emerges not merely through the perceptions of situations, but through appraisals of threats to goals and options available of dealing with threats. Our model expands upon previous studies by demonstrating that anxiety is situated within the goal-striving cycle.

A second contribution of our study is to provide a better understanding of the role of coping behaviors. We reveal and explain a wider range of previously unaccounted for coping responses. Moreover, we posit that the four coping categories revealed here specifically pertain to four points in an iterative goal-striving cycle. Consequently, our model goes significantly beyond static and binary conceptions of coping responses in entrepreneurship research, such as problem/emotion and approach/avoidance, which have been found inadequate in explaining higher-order coping responses (Lazarus, 1999; Skinner et al., 2003; Gross, 2015). For example, emotion-based coping should not be restricted to distraction or delaying (items we refer to as belonging to cat. 4), as argued by Patzelt and Shepherd (2011) and Uy et al. (2013), as other coping response categories also involve the regulation of emotion to influence the environment or change perceptions or goals (Gross, 2015). From the perspective of achieving goals and maintaining standards, all coping responses can potentially be both problem and emotion-focused, which explains the findings of Patzelt and Shepherd (2011) that the self-employed use both, and of Byrne and Shepherd (2015) that both are helpful in emotional regulation.

Furthermore, avoidance coping can help to solve problems; in fact, it is often intended to do just that. It is common that individuals temporarily district themselves from a situation so as to address it refreshed later on. Comparable to the finding by Folkman and Lazarus (1980), who found that people use both approach and avoidance coping in 98% of 1300 stressful episodes, the entrepreneurs in our sample also use both (if we assume coping responses pertaining to category 4 to be considered avoidance responses). Our study shows that category 4 responses are not simply about avoiding taking action, but rather explicitly intended to help facilitate category 1, 2, and 3 responses by gaining strength and reconsidering options. Conceiving of category 4 responses as being part of the goal-striving cycle, instead of existing in isolation, helps explain the findings of Uy et al. (2013) that entrepreneurs oscillate between and use both avoidance and approach coping, as well as the finding of Shepherd et al. (2009b) that entrepreneurs postpone quitting their venture until they are ready to quit (give up on the goal) (see also Rouse, 2016). That cat. 4 responses help individuals to gain or regain the strength to deal with the anxiety and its source helps to explain the finding by Uy et al. (2013) that avoidance coping has to be combined with active coping; cat. 4 responses by themselves do not close the gap but facilitate responses in the other categories which do.

More generally, our model and findings reinforce the point made by Uy et al. (2013) and Byrne and Shepherd (2015), as well as coping experts such as Skinner et al. (2003) and Folkman and Moskowitz (2004), that different coping responses are not inherently better or worse. The ways that individuals cope are assembled based on the specific situational demands and constraints, goal hierarchy and individual subjective preferences involved. Thus, any method of coping can be locally adaptive. This still leaves open the possibility that a particular coping response can prove ineffective in the long run, but this applies equally to what may be labeled as emotion or avoidance focused (e.g., cat. 4 response of habitually drinking alcohol) or problem and approach focused (e.g., cat. 1 response of bullying those perceived to be involved in creating the obstacle). Neither do we subscribe to the prescription for entrepreneurs to stay calm at all times, as He et al. (2018) maintain. For example, occasionally venting one’s emotions may very well help to solve issues as well as regulate emotions. Whether a coping response is effective, ultimately depends on whether it contributes to closing the goal-situation discrepancy.

A third contribution of our study is to go beyond fear of failure. Contrary to what one would expect based on fear of failure research, respondents talked in-depth about anxiety using the terms ‘doubt’ and ‘worry’ interchangeably, but seldom referred to ‘fear,’ ‘scared,’ or ‘afraid’ explicitly (in fact, several respondents strongly argued they were not afraid). Anxieties can concern immediate threats to business survival, similar to fear of failure, but also includes more opaque and lingering worries and doubts about making the right career choice, being a responsible person, maintaining or increasing self-esteem and reputation in the eyes of others, among others. The entrepreneurs in our study also indicated anxiety inducing emotions (e.g., frustration, anger, and loneliness) without stating they were fearful of outcomes (cf, Cope, 2011).

A fourth contribution of our study is to highlight the immediate beneficial activating effects of anxiety, as brought up by the majority of entrepreneurs interviewed (75%). This finding provides support for the position that, at least for active entrepreneurs, negative affect predominantly has positive direct effects on behavior and cognitive functioning. This is in line with the conceptual arguments of Cacciotti and Hayton (2015), as well as the empirical findings of Foo et al. (2009), Jennings et al. (2015), and Cacciotti et al. (2016). However, it contrasts with the findings of Doern and Goss (2014), Morgan and Sisak (2016), and the position of Shepherd (2015), who argues that a negative spiral effect beginning with a lack of progress generates negative emotions that again obstructs progress and so on. It is also in contrast with the findings of Kollmann et al. (2017), who report that obstacles provoke fear of failure, which elicits withdrawal and avoidance. Our explanation is that research participants uncommitted to the hypothetical lab situation in the study of Kollmann et al. (2017) may indeed quickly withdraw, whereas those in the field, who are committed to their ventures, will strive to persist. The majority of our respondents argued that anxieties actually enhanced their behavioral and cognitive functioning. In sum, for the entrepreneurs in our sample, threats resulted in efforts to reduce anxiety by striving to close discrepancies and achieve goals and standards, rather than giving up. Obviously, our sample is subject to survival bias, and the picture may well change if entrepreneurs who quit their venture are studied.

Finally, we find some evidence that entrepreneurs learn over time about the effectiveness of various responses in the four coping categories when repeatedly encountering the same source of anxiety. This reinforces the findings by Shepherd et al. (2011), Uy et al. (2013), Jenkins et al. (2014), and Cacciotti et al. (2016). For example, Uy et al. (2013) finding that experienced entrepreneurs make more effective use of avoidance strategies suggests that those entrepreneurs have learned to make more effective use of cat. 4 coping responses. Repeated successful efforts to cope with anxiety may build up resilience, or what Shepherd et al. (2009a) refer to as coping self-efficacy. Our respondents report that novel and immediate experiences regularly increase levels of anxiety. E.g., worries about capabilities or reliance may appear remote until a novel situation makes their anxiety inducing nature highly salient. Once the obstacle has been overcome, by use of any of the responses in the four categories, experience makes it easier deal with the anxiety when a similar situation resurfaces, or even prevents anxiety from arising. The latter effect provides an alternative explanation of Patzelt and Shepherd (2011) finding that the self-employed experience fewer emotions, which they attribute to a selection effect, but which can also be an effect of experience and learning.

### Limitations and Future Research

Our inductively derived conceptual understanding of anxiety and coping provides a basis for future research that can hopefully address the limitations to our study. First, our theoretical sampling method consists of selecting young, but surviving firms and thus, is open to survival bias. Given that many or most startups fail in the first five business years, many entrepreneurs in our sample will also fail. Therefore, our study reflects anxieties and coping behaviors of a mixture of entrepreneurs who will eventually succeed or fail. Having said that, we make no claims about the effectiveness of any one coping response for business survival and performance, as we do not track entrepreneurs over a long time period, thus are unable to make explicit comparisons among entrepreneurs who persist versus exit. Future studies will make progress by including recently failed ventures and looking for differences in responses about the motivational effects of anxiety and execution of coping responses. Moreover, we did not study decisions to persevere or to quit, like Kollmann et al. (2017). Coping strategies can also be seen as perseverance strategies as they allow entrepreneurs to persist with the venture (van Gelderen, 2012). Future research can look at selected cases to explore the configuration and sequence of coping responses in regard to decisions to halt or continue operations. Furthermore, future research seeking to develop measures of sources, immediate effects and coping responses pertaining to entrepreneurial anxiety may use our study to develop their initial item pool in efforts to increase generalizability and predictive statistical power.

Second, future studies may make headway exploring and explaining the configurations and sequences of coping responses to shine light on business survival during the entrepreneurial journey. As we have argued, the nature of anxiety and coping responses are subject to the goal hierarchy being pursued, which varies person to person. We have asked our respondents to take a helicopter view, particularly in wave 2, reflecting on how coping and anxiety develops over a long time period. To further justify our model, future research may also explore experience sampling methodologies (ESM), which require participants to provide reports of their thoughts, feelings, and behaviors associated with anxiety and coping at multiple times across situations as they happen in the field (Uy et al., 2010).

Third, future research may investigate the moderating role of personality attributes such as positive dispositional affect, which has been suggested to improve one’s ability to deal with anxiety (Baron, 2008; Baron et al., 2012; Podoynitsyna et al., 2012). Dispositional variables may influence the type of responses in the four coping categories adopted through either configuration and/or sequence. Studying personality attributes may also be relevant for future research looking at those who deliberately seek out anxiety. Future research may aim to provide a theoretical account for the behavioral, cognitive, motivational and emotional features of this group compared to those not actively seeking out anxiety. This is also linked to industry or sector; some industries are more uncertain and dynamic than others in which entrepreneurs require more resilience in order to succeed.

Fourthly, we studied how entrepreneurs respond to anxiety, and future research can study how entrepreneurs prevent anxiety from becoming overwhelming or from occurring at all. Research on resilience (Chadwick and Raver, 2018) and preventive coping (Reuter and Schwarzer, 2015), which concern the build-up of resources to deal with failure that may or may not occur in the future, may provide guidance here. One example of such a strategy is provided by Engel et al. (2019), who found that engagement in loving kindness meditation mitigates levels of fear of failure when confronted with a hypothetical aversive business situation. Another example is defensive pessimism (Norem, 2008): a combined strategy of setting low expectations (being pessimistic) and taking pre-emptive preventative steps with regard to the things that might go wrong as one prepares for an upcoming situation or task.

Finally, future research of particular interest is the study of serial/portfolio entrepreneurs in relation to various sources of anxiety. Jenkins et al. (2014) found that serial/portfolio entrepreneurs experience less grief, because autonomy, self-esteem and finances are still provided by concurrent or future businesses. In the context of our model, it means that a goal-involving response [cat. 3] of quitting does not result in termination of the goal-striving cycle, since this decision has to be seen in the wider context of the totality of goals involved. Future research can use the model presented in this article to study how entrepreneurs cope with anxiety while running single, as well as multiple business ventures.

### Practical Implications

Our study has practical implications for both aspiring and experienced entrepreneurs. Our study develops an awareness of the persistence of anxieties throughout the entrepreneurial journey. Anxiety does not just relate to business success/failure. Instead, the pursuit of entrepreneurial goals coincides with potential threats to a variety of goals, values, and standards. At the same time, our study shows that four different and interrelated categories of coping can be concurrently deployed to translate negative experiences into positive cognitive and behavioral effects, and that any response can be potentially effective. Nevertheless, since the entrepreneurial journey is dynamic and evolving, various anxieties will have more salience at different times, which implies a constant reconfiguration and maintenance of coping responses. As a result, continually building resilience to various and changing anxieties may be essentially what it means to be an entrepreneur.

## Conclusion

Organizational psychologists have an interest in entrepreneurship as it provides unique insight into human cognition and behavior under trying conditions (Baum et al., 2007). We used open-ended, vignette and graphical elicitation interviews with 77 entrepreneurs to investigate the nature, origins, and dynamics of anxiety and coping during the entrepreneurship process. We revealed ten sources, four categories of immediate effects, and four categories of coping responses of entrepreneurs. This then led to the development of a dynamic and comprehensive goal-oriented model of anxiety and coping. By doing so, we shed light on a range of the entrepreneurship literature on the self-regulation of negative emotions and open up a series of questions for future psychological research on the “emotional rollercoaster” of founding new organizations.

## Data Availability Statement

The datasets for this article are not publicly available to protect the privacy of the participants. Anonymized data are available on request to the corresponding author.

## Ethics Statement

Ethical review and approval was not required for the study on human participants in accordance with the local legislation and institutional requirements. Written informed consent for participation was not required for this study in accordance with the national legislation and the institutional requirements. The participants provided their informed consent to participate in this study, in verbal and/or written form.

## Author Contributions

All authors listed have made a substantial, direct and intellectual contribution to the work, and approved it for publication.

## Conflict of Interest

The authors declare that the research was conducted in the absence of any commercial or financial relationships that could be construed as a potential conflict of interest.

## References

[B1] Austin J. T. Vancouver J. B. (1996). Goal constructs in psychology: structure, process, and content. *Psychol. Bull.* 120 338–375. 10.1037/0033-2909.120.3.338

[B2] Barba-Sánchez V. Atienza-Sahuquillo C. (2017). Entrepreneurial motivation and self-employment: evidence from expectancy theory. *Int. Entrep. Manag. J.* 13 1097–1115. 10.1007/s11365-017-0441-z

[B3] Baron R. (2008). The role of affect in the entrepreneurial process. *Acad. Manag. Rev.* 33 328–340. 10.5465/amr.2008.31193166

[B4] Baron R. A. Frese M. Baum J. R. (2007). “Research gains: benefits of closer links between i/o psychology and entrepreneurship,” in *The Organizational Frontiers. The Psychology of Entrepreneurship*, eds Baum J. R. Frese M. Baron R. A. (Mahwah, NJ: Lawrence Erlbaum Associates Publishers), 347–373.

[B5] Baron R. A. Hmieleski K. M. Henry R. A. (2012). Entrepreneurs’ dispositional positive affect: the potential benefits - and potential costs - of being up. *J. Bus. Ventur.* 27 310–324. 10.1016/j.jbusvent.2011.04.002

[B6] Baum J. R. Frese M. Baron R. A. Katz J. A. (2007). “Entrepreneurship as an Area of Psychology Study: an Introduction,” in *The Psychology of Entrepreneurship The Organizational Frontiers*, eds Baum J. R. Frese M. Baron R. A. (Mahwah, NJ: Lawrence Erlbaum Associates Publishers), 1–18.

[B7] Baumeister R. F. Gailliot M. DeWall C. N. Oaten M. (2006). Self-regulation and personality: how interventions increase regulatory success, and how depletion moderates the effects of traits on behavior. *J. Pers.* 74 1773–1801. 17083666 10.1111/j.1467-6494.2006.00428.x

[B8] Benz M. Frey B. S. (2008). Being independent is a great thing: subjective evaluations of self-employment and hierarchy. *Economica* 75 362–383. 10.1111/j.1468-0335.2007.00594.x

[B9] Brooks J. King N. (2014). *Doing Template Analysis: Evaluating an End-of-Life Care Service.* Thousand Oaks, CA: SAGE.

[B10] Brooks J. McCluskey S. Turley E. King N. (2015). The Utility of Template Analysis in Qualitative Psychology Research. *Qual. Res. Psychol.* 12 202–222. 10.1080/14780887.2014.955224 27499705 PMC4960514

[B11] Byrne O. Shepherd D. A. (2015). Different strokes for different folks: entrepreneurial narratives of emotion, cognition, and making sense of business failure. *Entrep. Theory Pract.* 39 375–405. 10.1111/etap.12046

[B12] Cacciotti G. Hayton J. C. (2015). Fear and entrepreneurship: a review and research agenda. *Int. J. Manag. Rev.* 17 165–190. 10.1111/ijmr.12052

[B13] Cacciotti G. Hayton J. C. Mitchell J. R. Andres G. (2016). A reconceptualization of fear of failure in entrepreneurship. *J. Bus. Ventur.* 31 302–325. 10.1016/j.jbusvent.2016.02.002

[B14] Cardon M. S. Foo M.-D. Der Shepherd D. A. Wiklund J. (2012). Exploring the heart: entrepreneurial emotion is a hot topic. *Entrep. Theory Pract.* 36 1–10. 10.1111/j.1540-6520.2011.00501.x

[B15] Carver C. S. Scheier M. F. (1981). *Attention and Self-Regulation: A Control-Theory Approach to Human Behavior.* Berlin: Springer.

[B16] Carver C. S. Scheier M. F. (1998). *On the Self-Regulation of Behavior.* Cambridge: Cambridge University Press.

[B17] Chadwick I. C. Raver J. L. (2018). Psychological resilience and its downstream effects for business survival in nascent entrepreneurship. *Entrep. Theory Pract.* 44, 233–255.

[B18] Cope J. (2011). Entrepreneurial learning from failure: an interpretative phenomenological analysis. *J. Bus. Ventur.* 26 604–623. 10.1016/j.jbusvent.2010.06.002

[B19] Cope J. Watts G. (2000). Learning by doing – An exploration of experience, critical incidents and reflection in entrepreneurial learning. *Int. J. Entrep. Behav. Res.* 6 104–124. 10.1108/13552550010346208

[B20] Creswell J. W. Miller D. L. (2000). Determining validity in qualitative inquiry. *Theory Pract.* 39 124–130. 10.1207/s15430421tip3903_2

[B21] Crilly N. Blackwell A. F. Clarkson P. J. (2006). Graphic elicitation: using research diagrams as interview stimuli. *Qual. Res.* 6 341–366. 10.1177/1468794106065007

[B22] De Cock R. Denoo L. Clarysse B. (2019). Surviving the emotional rollercoaster called entrepreneurship: the role of emotion regulation. *J. Bus. Ventur.* (in press).

[B23] Dess G. (2003). Emerging issues in corporate entrepreneurship. *J. Manag.* 29 351–378. 10.1016/s0149-2063(03)00015-1

[B24] Doern R. Goss D. (2014). The role of negative emotions in the social processes of entrepreneurship: power rituals and shame-related appeasement behaviors. *Entrep. Theory Pract.* 38 863–890. 10.1111/etap.12026

[B25] Ekore J. O. Okekeocha O. C. (2012). Fear of entrepreneurship among university graduates: a psychological analysis. *Int. J. Manag.* 29 515–525.

[B26] Engel Y. Noordijk S. Spoelder A. van Gelderen M. (2019). Self-compassion when coping with venture obstacles: loving-kindness meditation and entrepreneurial fear of failure. *Entrep. Theory Pract.* (in press).

[B27] Fodor O. C. Pintea S. (2017). The “emotional side” of entrepreneurship: a meta-analysis of the relation between positive and negative affect and entrepreneurial performance. *Front. Psychol.* 8:310. 10.3389/fpsyg.2017.00310 28348534 PMC5346553

[B28] Folkman S. Lazarus R. S. (1980). An analysis of coping in a middle-aged community sample. *J. Health Soc. Behav.* 21:219 10.2307/2136617 7410799

[B29] Folkman S. Moskowitz J. T. (2004). Coping: pitfalls and promise. *Annu. Rev. Psychol.* 55 745–774. 10.1146/annurev.psych.55.090902.141456 14744233

[B30] Foo M. D. Uy M. A. Baron R. A. (2009). How do feelings influence effort? An empirical study of entrepreneurs’ affect and venture effort. *J. Appl. Psychol.* 94 1086–1094. 10.1037/a0015599 19594247

[B31] Foo M. D. Uy M. A. Murnieks C. (2015). Beyond affective valence: untangling valence and activation influences on opportunity identification. *Entrep. Theory Pract.* 39 407–431. 10.1111/etap.12045

[B32] Gollop R. Whitby E. Buchanan D. Ketley D. (2004). Influencing sceptical staff to become supporters of service improvement: a qualitative study of doctors’ and managers’ views. *Qual. Saf. Heal. Care* 13 108–114. 10.1136/qshc.2003.007450 15069217 PMC1743804

[B33] Grégoire D. A. Cornelissen J. P. Dimov D. van Burg E. (2015). The mind in the middle: taking stock of affect and cognition research in entrepreneurship. *Int. J. Manag. Rev.* 17 125–142. 10.1111/ijmr.12060

[B34] Grichnik D. Smeja A. Welpe I. (2010). The importance of being emotional: how do emotions affect entrepreneurial opportunity evaluation and exploitation? *J. Econ. Behav. Organ.* 76 15–29. 10.1016/j.jebo.2010.02.010

[B35] Gross J. J. (2015). Emotion regulation: current status and future prospects. *Psychol. Inq.* 26 1–26. 10.1254/fpj.151.21 29321392

[B36] Guest G. MacQueen K. M. Namey E. E. (2012). *Applied Thematic Analysis.* Thousand Oaks, CA: SAGE Publications.

[B37] Hayton J. C. Cholakova M. (2011). The role of affect in the creation and intentional pursuit of entrepreneurial ideas. *Entrep. Theory Pract.* 36 41–68. 10.1111/j.1540-6520.2011.00458.x

[B38] He V. F. Sirén C. Singh S. Solomon G. (2018). Keep calm and carry on: emotion regulation in entrepreneurs’ learning from failure. *Entrep. Theory Pract.* 42 605–630. 10.1177/1042258718783428

[B39] Holland D. V. Garrett R. P. (2015). Entrepreneur start-up versus persistence decisions: a critical evaluation of expectancy and value. *Int. Small Bus. J. Res. Entrep.* 33 194–215. 10.1177/0266242613480375

[B40] Jenkins A. S. Wiklund J. Brundin E. (2014). Individual responses to firm failure: appraisals, grief, and the influence of prior failure experience. *J. Bus. Ventur.* 29 17–33. 10.1016/j.jbusvent.2012.10.006

[B41] Jennings J. E. Edwards T. Devereaux Jennings P. Delbridge R. (2015). Emotional arousal and entrepreneurial outcomes: combining qualitative methods to elaborate theory. *J. Bus. Ventur.* 30 113–130. 10.1016/j.jbusvent.2014.06.005

[B42] Kaplan A. Maehr M. L. (2007). The contributions and prospects of goal orientation theory. *Educ. Psychol. Rev.* 19 141–184. 10.1007/s10648-006-9012-5

[B43] Kehr H. M. (2004). Integrating implicit motives, explicit motives, and perceived abilities: the compensatory model of work motivation and volition. *Acad. Manag. Rev.* 29 479–499. 10.5465/amr.2004.13670963

[B44] Kent G. (2000). Understanding the experiences of people with disfigurements: an integration of four models of social and psychological functioning. *Psychol. Heal. Med.* 5 117–129. 10.1080/713690187 29156953

[B45] King N. Brooks J. M. (2017). *Template Analysis for Business and Management Students.* London: SAGE Publications.

[B46] Kollmann T. Stöckmann C. Kensbock J. M. (2017). Fear of failure as a mediator of the relationship between obstacles and nascent entrepreneurial activity—an experimental approach. *J. Bus. Ventur.* 32 280–301. 10.1016/j.jbusvent.2017.02.002

[B47] Lazarus R. S. (1966). *Psychological Stress and the Coping Process.* New York, NY: McGraw-Hill.

[B48] Lazarus R. S. (1996). “The role of coping in the emotions and how coping changes over the life course,” in *Handbook of Emotion, Adult Development, and Aging*, eds Maletesta-Magni C. McFadden S. H. (New York: Academic Press), 289–306. 10.1016/b978-012464995-8/50017-0

[B49] Lazarus R. S. (1999). *Stress and Emotion.* New York, NY: Springer.

[B50] Lazarus R. S. Folkman S. (1984). *Stress, Appraisal, and Coping.* New York, NY: Springer.

[B51] Miceli M. Castelfranchi C. (2005). Anxiety as an “epistemic” emotion: an uncertainty theory of anxiety. *Anxiety, Stress Coping* 18 291–319. 10.1080/10615800500209324

[B52] Millán J. M. Congregado E. Román C. (2012). Determinants of self-employment survival in Europe. *Small Bus. Econ.* 38 231–258. 10.1007/s11764-011-0183-9 21681406

[B53] Morgan J. Sisak D. (2016). Aspiring to succeed: a model of entrepreneurship and fear of failure. *J. Bus. Ventur.* 31 1–21. 10.1016/j.jbusvent.2015.09.002

[B54] Morris M. H. Kuratko D. F. Schindehutte M. Spivack A. J. (2012). Framing the entrepreneurial experience. *Entrep. Theory Pract.* 36 11–40. 10.1037/apl0000437 31343203

[B55] Norem J. K. (2008). Defensive pessimism, anxiety, and the complexity of evaluating self-regulation. *Soc. Personal. Psychol. Compass* 2 121–134. 10.1111/j.1751-9004.2007.00053.x

[B56] Parslow R. A. Jorm A. F. Christensen H. Bryan R. (2004). The associations between work stress and mental health: a comparison of organizationally employed and self-employed workers. *Work Stress* 18 231–244. 10.1080/14749730412331318649

[B57] Patzelt H. Shepherd D. A. (2011). Negative emotions of an entrepreneurial career: self-employment and regulatory coping behaviors. *J. Bus. Ventur.* 26 226–238. 10.1016/j.jbusvent.2009.08.002

[B58] Podoynitsyna K. Van der Bij H. Song M. (2012). The role of mixed emotions in the risk perception of novice and serial entrepreneurs. *Entrep. Theory Pract.* 36 115–140. 10.1111/j.1540-6520.2011.00476.x

[B59] Poppleton S. Briner R. B. Kiefer T. (2008). The roles of context and everyday experience in understanding work-non-work relationships: a qualitative diary study of white- and blue-collar workers. *J. Occup. Organ. Psychol.* 81 481–502. 10.1348/096317908x295182

[B60] Rauch A. Fink M. Hatak I. (2018). Stress processes: an essential ingredient in the entrepreneurial process. *Acad. Manag. Learn. Educ.* 32 340–357. 10.5465/amp.2016.0184

[B61] Reuter T. Schwarzer R. (2015). “Manage stress at work through preventive and proactive coping,” in *Handbook of Principles of Organizational Behavior*, ed. Locke E. A. (Hoboken, NJ: WILEY-BLACKWELL), 499–515. 10.1002/9781119206422.ch27

[B62] Rouse E. D. (2016). Beginning’s end: how founders psychologically disengage from their organizations. *Acad. Manag. J.* 59 1605–1629. 10.5465/amj.2013.1219

[B63] Schindehutte M. Morris M. Allen J. (2006). Beyond achievement: entrepreneurship as extreme experience. *Small Bus. Econ.* 27 349–368. 10.1007/s11187-005-0643-6

[B64] Shepherd D. A. (2015). Party On! A call for entrepreneurship research that is more interactive, activity based, cognitively hot, compassionate, and prosocial. *J. Bus. Ventur.* 30 489–507. 10.1016/j.jbusvent.2015.02.001

[B65] Shepherd D. A. Covin J. G. Kuratko D. F. (2009a). Project failure from corporate entrepreneurship: managing the grief process. *J. Bus. Ventur.* 24 588–600. 10.1016/j.jbusvent.2008.01.009

[B66] Shepherd D. A. Patzelt H. Wolfe M. (2011). Moving forward from project failure: negative emotions, affective commitment, and learning from the experience. *Acad. Manag. J.* 54 1229–1212.

[B67] Shepherd D. A. Wiklund J. Haynie J. M. (2009b). Moving forward: balancing the financial and emotional costs of business failure. *J. Bus. Ventur.* 24 134–148. 10.1016/j.jbusvent.2007.10.002

[B68] Sitkin S. B. (1992). “Learning through failure: the strategy of small losses,” in *Research in Organizational Behavior*, eds Cummings L. L. Staw B. M. (Des Moines, IA: Department of Management), 231–266.

[B69] Skinner E. A. Edge K. Altman J. Sherwood H. (2003). Searching for the structure of coping: a review and critique of category systems for classifying ways of coping. *Psychol. Bull. V* 129 216–269. 10.1037/0033-2909.129.2.216 12696840

[B70] Stephan U. (2018). Entrepreneurs’ mental health and well-being: a review and research agenda. *Acad. Manag. Perspect.* 32 290–322. 10.5465/amp.2017.0001

[B71] Stephan U. Roesler U. (2010). Health of entrepreneurs versus employees in a national representative sample. *J. Occup. Organ. Psychol.* 83 717–738. 10.1348/096317909x472067

[B72] Uy M. A. Foo M.-D. Song Z. (2013). Joint effects of prior start-up experience and coping strategies on entrepreneurs’ psychological well-being. *J. Bus. Ventur.* 28 583–597. 10.1016/j.jbusvent.2012.04.003

[B73] Uy M. A. Foo M. D. D. Aguinis H. (2010). Using experience sampling methodology to advance entrepreneurship theory and research. *Organ. Res. Methods* 13 31–54. 10.1177/1094428109334977

[B74] van Gelderen M. (2012). Perseverance strategies of enterprising individuals. *Int. J. Entrep. Behav. Res.* 18 630–648. 10.1108/13552551211268102

[B75] van Gelderen M. (2016). Entrepreneurial autonomy and its dynamics. *Appl. Psychol.* 65 541–567. 10.1016/j.socscimed.2012.03.055 22655672

[B76] Vancouver J. B. Day D. V. (2005). Industrial and organisation research on self-regulation: from constructs to applications. *Appl. Psychol.* 54 155–185. 10.1111/j.1464-0597.2005.00202.x

[B77] Welpe I. M. Spörrle M. Grichnik D. Audretsch D. B. (2012). Emotions and opportunities: the interplay of opportunity evaluation, fear, joy, and anger as antecedent of entrepreneurial exploitation. *Entrep. Theory Pract.* 36 69–96. 10.1111/j.1540-6520.2011.00481.x

