# Supplementary material for: No Need to Worry? Anxiety and Coping in the Entrepreneurship Process
Source: Front Psychol. 2020 Mar 12;11:398. doi: 10.3389/fpsyg.2020.00398 (PMC7080856; doi:10.3389/fpsyg.2020.00398)
Supplement: Supplementary file 1 [file Data_Sheet_1.docx]

**APPENDIX 1**

**Vignettes Used in Second Wave Data Collection and Analysis**

| **Business model viability** | Tom is launching a new product next week for his new company. He is afraid that the product will not be taken seriously by customers and clients. He knows that there is a chance that the product will not work properly. He is also worried that his social media marketing campaign may fail. He worries about the viability of the business concept. |
| --- | --- |
| **Growth** | Bart is an entrepreneur who aims to grow his company. He has managed to get the business started and derive some regular revenue, but he now needs to take it to the next level. He worries about whether he can obtain more revenue and find new customers and clients. He has an ambitious plan to grow his venture, but this requires considerable financial investment of his savings, and he worries about losing the money. |
| **Opportunity costs** | Tim had the choice between joining a marketing department of an established fashion corporation, or starting his own marketing firm. He chose to start his own company. He also has taken a mortgage on his home and investing most of his savings, as well as a great deal of time and energy. But then, he worries that he may have missed out on the corporate opportunity. Tim is also anxious that his hard work means that he spends less time with his young son, wife, and friends. |
| **Capability** | Fred started a web-based services company. However, often he finds himself a bit out of his depth, having to tackle situations and problems that he does not have much experience with. Sometimes he doesn’t really know what to do and can’t come up with any ideas, causing him considerable anxiety. He often doubts the decisions he makes, feeling he is not good at making good decision, causing him to always second guess himself. |
| **Dependence** | Joost has started a new technology company and has sold enough retail product to hire employees and gain an investor. However, he often feels anxious that the people he hired do not have the best skills and commitment to the project. He worries that one of the employees will leave suddenly as they are redeveloping the product. Moreover, his investor has yet to transfer the final sum of money that Joost needs to pay his employees, and one client is having financial difficulties and may not be able to pay for a recent order. |
| **Responsibility** | John and Eve always dreamed of starting their own restaurant. They borrowed money from a number of good friends and relatives to make this dream come true. They have hired a number of excellent employees, and just completed a catering contract with a large corporation for an upcoming event. John feels highly responsible towards his investors, employees, and co-founder, and often feels anxious that the restaurant may not become a success and that he will let them down. He is also worried that he has promised too much, and will not be able to complete the catering contract on time. |
| **Environmental uncertainty** | Vera is an entrepreneur in the creative industries. She likes to be in control, yet the market is highly dynamic and unpredictable. She spends a lot of time planning, but new developments arise every week or month. New competitors are often entering the market, many of them replicating her company’s business model. The lack of control she feels makes her feel anxious. |
| **Finance** | Paul and a co-founder are starting a gaming software company. Both entrepreneurs are excited and confident about the opportunity and they spend much of their time with the product. Still having few paying customers, invoices addressed to the company pile up; Paul is now also starting to feel anxious about his private financial matters and is worried about being able to pay rent and other bills, and his quickly disappearing savings. |
| **Social appraisal** | Bart is an entrepreneur who runs a fast growing company. He has often felt anxious about the expectations that people have of him, as he feels he should live up to their expectations of being highly successful. This anxiety has recently increased after having had a bit of exposure in the media and winning a prize for being his city’s entrepreneur of the year. |
| **Self-appraisal** | Shanna is a young entrepreneur who started a retail business whilst finishing a master’s degree. She has high standards for herself as an entrepreneur and sees herself as a determined personality who generally succeeds at whatever she wants to accomplish. However, she now starts to worry about the business not being successful, which she would regard as a personal failure which diminishes her sense of self-worth. |
